# Investigation of Recurrent Melioidosis in Lao People's Democratic Republic by Multilocus Sequence Typing

**DOI:** 10.4269/ajtmh.15-0909

**Published:** 2016-06-01

**Authors:** Audrey Rachlin, Sabine Dittrich, Koukeo Phommasone, Anousone Douangnouvong, Rattanaphone Phetsouvanh, Paul N. Newton, David A. B. Dance

**Affiliations:** London School of Hygiene and Tropical Medicine, London, United Kingdom; Lao-Oxford-Mahosot Hospital-Wellcome Trust Research Unit, Microbiology Laboratory, Mahosot Hospital, Vientiane, Lao People's Democratic Republic; Centre for Tropical Medicine and Global Health, University of Oxford, Oxford, United Kingdom

## Abstract

Melioidosis is an infectious disease caused by the saprophytic bacterium *Burkholderia pseudomallei*. In northeast Thailand and northern Australia, where the disease is highly endemic, a range of molecular tools have been used to study its epidemiology and pathogenesis. In the Lao People's Democratic Republic (Laos) where melioidosis has been recognized as endemic since 1999, no such studies have been undertaken. We used a multilocus sequence typing scheme specific for *B. pseudomallei* to investigate nine cases of culture-positive recurrence occurring in 514 patients with melioidosis between 2010 and 2015: four were suspected to be relapses while the other five represented reinfections. In addition, two novel sequence types of the bacterium were identified. The low overall recurrence rates (2.4%) and proportions of relapse and reinfection in the Laos are consistent with those described in the recent literature, reflecting the effective use of appropriate antimicrobial therapy.

The gram-negative bacterium *Burkholderia pseudomallei* is the cause of melioidosis, an infectious disease endemic throughout the tropics, especially southeast Asia and northern Australia. Case fatality rates range between 14% and 40%, even with appropriate treatment, and recurrence is a potential problem in those who survive.^1^,^2^ In the past, recurrence was found to occur in as many as 13–23% of melioidosis patients, with genotyping of initial and recurrent isolates suggesting that most of these were relapses due to failure to eradicate the initial infecting strain.^2^–^4^ The use of longer courses of treatment in countries such as Thailand and Australia has dramatically reduced the overall recurrence rate to as low as 1.2–7%.^1^,^4^ Furthermore, the proportion of recurrences attributed to reinfection as opposed to relapse has increased.^1^,^4^,^5^

Melioidosis was first described in the Lao People's Democratic Republic (Laos) in 1999 but is now known to be highly endemic, with more than 920 culture-positive cases diagnosed by the Microbiology Laboratory of Mahosot Hospital, Vientiane, in the past 15 years (unpublished data). Despite problems of affordability,^6^ international consensus guidelines for treatment are usually followed, comprising ceftazidime for at least 10–14 days followed by co-trimoxazole for 12–20 weeks (combined with doxycycline, before the publication of the MERTH study in 2014).^1^,^6^,^7^

Multilocus sequence typing (MLST) has emerged as an effective tool to differentiate between relapses and reinfections in melioidosis.^3^,^8^ We have reviewed the incidence of recurrence in Lao patients and used MLST to investigate whether these cases were attributable to relapse or reinfection.

All patients from whom *B. pseudomallei* was isolated between 1999 and 2015 in the Microbiology Laboratory of Mahosot Hospital were recorded in a database. Bacteria were isolated during studies of the etiology of fever in Laos, and isolates were stored at −80°C. Ethical approval for the study was obtained from the Oxford Tropical Research Ethics Committee and The National Ethics Committee for Health Research, Laos. Culture-confirmed cases occurring after January 1, 2010, were included in the analysis. Recurrence was defined as the development of symptoms compatible with melioidosis after completion of treatment, associated with the isolation of *B. pseudomallei* from any clinical sample.

Genomic DNA was isolated using the Qiagen Mini Kit (Hilden, Germany) according to the manufacturer's instructions. Seven housekeeping alleles were amplified by polymerase chain reaction (PCR) for each isolate using the described MLST scheme.^9^ PCR products were analyzed on a 1.5% agarose gel (Seakem LE Agarose, Lonza, Visp, Switzerland) and, depending on purity, target amplicons were either processed by ethanol purification or gel extraction (QIAquick; Qiagen) and sequenced (Macrogen, Korea) on both forward and reverse strands. Only high-quality consensus sequences were subsequently used for final analysis using CLC Genomics Workbench 7.0 software (Qiagen). Consensuses were edited to the appropriate allele length before being entered into the MLST online database (available at: http://pubmlst.org/bpseudomallei/) for allele number assignment. Allele numbers were compiled into a series of seven integers corresponding to the gene order of *ace-gltB-gmhD-lepA-lipA-narK-ndh*, giving an allele profile for each isolate. The allele profiles were queried against the *B. pseudomallei* MLST website to obtain a sequence type (ST). The Fisher's exact test was used to assess categorical variables using Stata software (College Station, TX), and a *P* value < 0.05 was considered statistically significant.

Overall, 370 of 514 (72%) patients admitted after January 1, 2010, survived to be discharged from the hospital, and among these, nine (9/370, 2.4%) had culture-positive recurrence. Initial and recurrent isolates from all nine patients were available for analysis, and details of the patients and isolates are given in Table 1. The nine patients were between 2 and 63 (median: 42.4) years of age at the time of initial hospitalization, and the majority (6/9, 66.7%) were from Vientiane Capital or the adjacent Vientiane Province. Nearly all patients (7/9, 77.8%) had an underlying medical condition or occupation that would put them at a greater risk for infection, most notably farming (3/9, 33.3%) and diabetes mellitus (6/9, 66.7%), or both (3/9, 33.3%). Recurrent melioidosis was fatal in only one of the nine patients (MM384). In four cases (including two of the patients classified as relapses) ceftazidime was given for longer than 10–14 days often recommended during their initial illness, reflecting slow fever clearance and multifocal disease.

MLST profiles were assigned to all primary and secondary isolates (Table 2). On the basis of the MLST results, four of the nine patients, representing 0.78% (4/514) of all culture-confirmed melioidosis cases, were identified as probable relapses, as the STs of the primary and recurrent isolates were identical. The time to recurrence was between 2 and 32 (median: 13.5) months (Table 1). One of these (MM476) presented with symptoms and signs of recurrence while still on treatment with oral co-amoxiclav, although his adherence was uncertain, and is thus best considered as a case of recrudescence. Five patients (5/9, 55.5%) had initial and recurrent isolates with different STs, suggesting reinfection. The time to recurrence for these five patients was between 13 and 47 (median: 24.6) months. The median times to relapse and to reinfection were not significantly different (*P* = 0.17), but the number of cases analyzed was small. Two of the isolates, MM360.2 and MM452.2, had novel allele profiles and were subsequently assigned as ST1428 and ST1429, by the MLST database curator, respectively.

The results of this study provide evidence for a low recurrence rate (2.4%) of melioidosis in Laos, consistent with recent literature from other countries.^5^ Our data also suggest that, as elsewhere, recurrent melioidosis in this small number of cases from Laos was more frequently due to reinfection than relapse.^3^–^5^ This reflects the fact that considerable efforts have been made to implement international consensus antimicrobial therapy for melioidosis in Laos.

Although it is possible that some patients with recurrent infections might have been missed, for example, if they had died at home or presented to other hospitals where melioidosis could not be diagnosed by culture, great attention was paid ensuring compliance with treatment and with follow-up. The risk of relapse and importance of completing a full course of eradication treatment was repeatedly stressed to the patients and their family whenever possible, or their physicians when they were in hospitals other than Mahosot Hospital. On the completion of intensive phase treatment, patients were given a card describing the treatment plan and were asked to return regularly for follow-up at Mahosot Hospital, although this was not possible for the three patients (MM439, MM452, and MM562) under treatment in other hospitals. During follow-up visits, patients were asked about adherence, although this was not formally assessed, for example, by testing urinary antimicrobial activity. Antibiotics were provided free of charge to individuals not able to afford them. Consequently, we do not believe that access to medicines is likely to have been a significant problem.

*Burkholderia pseudomallei* has been shown to possess a high degree of strain diversity in both Thailand and northern Australia.^10^–^12^ Consistent with these findings, 13 different STs were identified among the 18 isolates from Laos, including two novel STs. The high degree of diversity makes it likely that that the four cases of recurrence we identified with the same ST are genuine relapses, although reinfection by the same strain cannot be completely ruled out.

Similarly, we assumed that individual infections were not caused by multiple *B. pseudomallei* strains, although polyclonal infections have occasionally been identified in melioidosis patients.^13^,^14^ If only a single ST were isolated and saved during a primary mixed infection, relapse could still have occurred with a different ST that had been present initially. In addition, if a polyclonal infection were to have occurred in one of these patients, it could have resulted in recombination between bacterial strains. Though unlikely, this may have led to modifications within an allele fragment, enough to warrant it being assigned a new allele number.^14^,^15^ Finally, microevolution occurring during the course of infection might occasionally result in a change of ST.^15^ All of these mechanisms could have resulted in relapses being wrongly classified as reinfections. However, since polyclonal infection does not appear to be common, and since none of our recurrent isolates were single locus variants of the initial strain, we consider this to be relatively unlikely.^16^

Collectively, our data suggest that appropriate antimicrobial therapy is being used in Laos for the treatment of melioidosis and that effective treatment of *B. pseudomallei* is possible in resource-limited settings if evidence-based treatment regimens are used. Studies utilizing higher resolution molecular indexing tools, such as whole-genome sequencing, will help to clarify whether this breakdown into relapse and reinfection is correct and assist with further epidemiological studies of melioidosis in Laos.

## Figures and Tables

**Table 1:** Sample and clinical information for the nine patients with recurrent melioidosis

Patient ID	Description	Admission date	Age (years)	Sex	Sample type	Home address (village, district, province)	Underlying conditions	Occupation	Outcome	Days IV ceftazidime
MM321	Initial infection	April 5, 2010	50	M	Hemoculture	Nongvaeng, Hadxaifong, Vientiane Capital	Hypertension and blood dyscrasia	Construction worker	Discharged healthy	32
MM321.2	Recurrent infection	December 9, 2010	50	M	Pus	Nongvaeng, Hadxaifong, Vientiane Capital	Hypertension and blood dyscrasia	Construction worker	Discharged healthy	22
MM360	Initial infection	August 4, 2010	45	M	Hemoculture	Xokgnai, Xaysettha, Vientiane Capital	Diabetes	Farmer	Discharged healthy	12
MM360.2	Recurrent infection	September 24, 2014	49	M	Hemoculture	Xokgnai, Xaysettha, Vientiane Capital	Diabetes	Farmer	Discharged healthy	21
MM363	Initial infection	August 13, 2010	59	F	Hemoculture	Nonsavang, Viengkham, Vientiane Province	Diabetes and gastric ulcer	Cook	Discharged healthy	27
MM363.2	Recurrent infection	June 20, 2012	61	F	Hemoculture	Nonsavang, Viengkham, Vientiane Province	Diabetes	Cook	Discharged healthy	21
MM384	Initial infection	September 5, 2010	43	M	Hemoculture	Phonkham, Phonhong, Vientiane Province	Diabetes	Farmer	Discharged healthy	33
MM384.2	Recurrent infection	June 1, 2013	46	M	Hemoculture	Phonkham, Phonhong, Vientiane Province	Diabetes	Farmer	Death	3
MM439	Initial infection	April 2, 2011	10	M	Pus	Phonsim, Kaysone, Savannakhet	None known	Student	Discharged healthy	10
MM439.2	Recurrent infection	May 23, 2012	11	M	Pus	Phonsim, Kaysone, Savannakhet	None known	Student	Discharged healthy	12
MM452	Initial infection	June 14, 2011	55	M	Hemoculture	Nonborkell, Xaysettha, Vientiane Capital	Diabetes and renal calculi	Merchant	Discharged healthy	14
MM452.2	Recurrent infection	January 21, 2013	57	M	Hemoculture	Nonborkell, Xaysettha, Vientiane Capital	Diabetes and renal calculi	Merchant	Discharged healthy	20
MM476	Initial infection	September 1, 2011	2	M	Throat Swab	Chansavang, Sikhottabong, Vientiane Capital	None known	Child	Discharged healthy	10
MM476.2	Recurrent infection	November 2, 2011	3	M	Pus	Chansavang, Sikhottabong, Vientiane Capital	None known	Child	Discharged healthy	10
MM545	Initial infection	September 21, 2012	60	M	Hemoculture	Huayxai, Pakxanh, Bolikhamxay	Diabetes	Farmer	Discharged healthy	32
MM545.2	Recurrent infection	June 12, 2014	63	M	Hemoculture	Huayxai, Pakxanh, Bolikhamxay	Diabetes, cirrhosis, renal failure	Farmer	Discharged healthy	14
MM562	Initial infection	November 18, 2012	49	M	Hemoculture	Lak, Salavan, Salavan	None known	Government official	Discharged healthy	14
MM562.2	Recurrent infection	November 22, 2013	50	M	Hemoculture	Lak, Salavan, Salavan	Diabetes	Government official	Discharged healthy	10

F = female; IV = intravenous; M = male. Recurrences have been designated with “.2” in sample IDs.

**Table 2:** Allele profiles and STs for the 18 isolates as assigned by the online *Burkholderia pseudomallei* multilocus sequence typing database (http://bpseudomallei.mlst.net/)

Sample ID	*ace*	*gltB*	*gmhD*	*lepA*	*lipA*	*narK*	*ndh*	ST
MM321	3	1	2	2	1	11	1	ST52
MM321.2	3	1	2	2	1	11	1	ST52
MM360	3	1	4	1	1	4	1	ST56
MM360.2	1	4	13	2	1	11	1	ST1428*
MM363	1	1	2	1	6	4	1	ST1004
MM363.2	1	1	3	3	1	2	1	ST488
MM384	3	1	4	3	1	4	3	ST375
MM384.2	3	1	4	3	1	4	3	ST375
MM439	1	4	2	3	8	4	3	ST376
MM439.2	1	4	2	1	1	4	3	ST658
MM452	1	1	13	1	1	1	1	ST10
MM452.2	1	4	13	1	1	1	3	ST1429*
MM476	3	1	4	1	1	4	1	ST56
MM476.2	3	1	4	1	1	4	1	ST56
MM545	1	2	3	1	1	3	1	ST307
MM545.2	3	1	11	3	5	4	6	ST507
MM562	3	4	11	3	5	4	6	ST70
MM562.2	3	4	11	3	5	4	6	ST70

STs = sequence types. Recurrent infections have been designated with “.2” in sample IDs.

* ST first described in this publication.
